# EffiSegNet: Advances in Automated Segmentation of Actinic Cheilitis and Lip Squamous Cell Carcinoma in Clinical Photographs

**DOI:** 10.1111/jop.70176

**Published:** 2026-08-23

**Authors:** Vitor Simbalista Teixeira Soares, Anna Luíza Damaceno Araújo, Ivan José Correia‐Neto, Cristina Saldivia‐Siracusa, Raísa Sales de Sá, Hugo Abritta Reis, Ayrton da Costa Ganem Filho, André Carlos Ponce de Leon Ferreira de Carvalho, Thierry Urruty, Paulo Rogério Ferreti Bonan, Nathalia Rodrigues Gomes, Felipe Paiva Fonseca, Pablo Agustin Vargas, Alan Roger Santos‐Silva, Luiz Paulo Kowalski, Matheus Cardoso Moraes, Marcio Ajudarte Lopes

**Affiliations:** ^1^ Institute of Science and Technology (ICT‐UNIFESP) Federal University of São Paulo São José dos Campos São Paulo Brazil; ^2^ Head and Neck Surgery Department University of São Paulo Medical School (FMUSP) São Paulo Brazil; ^3^ Hospital Israelita Albert Einstein São Paulo Brazil; ^4^ Departamento de Diagnóstico Oral, Faculdade de Odontologia de Piracicaba Universidade Estadual de Campinas (FOP‐UNICAMP) Piracicaba São Paulo Brazil; ^5^ School of Dentistry Ceuma University São Luís Brazil; ^6^ Institute of Mathematics and Computer Sciences of University of São Paulo (ICMC‐USP) São Paulo Brazil; ^7^ Université de Poitiers Univ. Limoges, CNRS, XLIM Poitiers France; ^8^ I3M Common Laboratory CNRS‐Siemens Poitiers France; ^9^ Department of Clinical and Social Dentistry, Health Sciences Center Federal University of Paraíba‐UFPB João Pessoa Brazil; ^10^ Department of Oral Surgery and Pathology, School of Dentistry Federal University of Minas Gerais (UFMG) Belo Horizonte Minas Gerais Brazil; ^11^ Department of Head and Neck Surgery and Otorhinolaryngology A.C. Camargo Cancer Center São Paulo Brazil

**Keywords:** actinic cheilitis, artificial intelligence, convolutional neural network, deep learning, lip squamous cell carcinoma, segmentation

## Abstract

**Background:**

Actinic cheilitis (AC) is a potentially malignant oral disorder linked to lip squamous cell carcinoma (LSCC). Clinical diagnosis is hindered by lesion heterogeneity. While AI tools have shown promise in oral lesion detection and diagnosis, image segmentation remains underexplored. This study developed and externally validated a deep learning segmentation model for digital photographs of lip lesions.

**Methods:**

A total of 489 clinical photographs from one institution were used for training/internal testing, and 82 external images from two independent institutions (UFPB and UFMG) served as external validation. EffiSegNet‑B0 used Image Net‑pretrained EfficientNet‑B0 encoder, with stage features aggregated via Ghost Modules. Manual segmentation masks comprised the clinically affected lip region and were used as ground truth. Performance was assessed using accuracy, sensitivity (recall), precision, Dice Similarity Coefficient (DSC) and intersection over union (IoU).

**Results:**

The model achieved high accuracy (> 95%) on both internal and external datasets. DSCs were 0.888 (95% CI: 0.871–0.904) for internal test and 0.875 (95% CI: 0.862–0.888) for external validation, while IoU values were 0.802 (95% CI: 0.777–0.828) and 0.782 (95% CI: 0.763–0.801), respectively. No performance decline was observed on external validation data. The model remained robust to lesion variations (ulceration, crusting, and keratinization).

**Conclusion:**

EffiSegNet‐B0 demonstrated high accuracy and adaptability in segmenting lip lesions, reinforcing its potential for clinical image analysis applications. The proposed model generalizes well across different institutions and lesion phenotypes, consistently extracting the complete region of interest. This proof‐of‐concept study supports its promise as a tool for automated lip lesion segmentation in heterogeneous clinical settings.

## 1 Introduction

Actinic cheilitis (AC) is an oral potentially malignant disorder that affects the vermilion of the lip, often preceding the development of lip squamous cell carcinoma (LSCC). Its main cause is prolonged exposure to ultraviolet radiation. The clinical diagnosis of AC is challenging due to the great variability in the presentation of the lesions, which can range from irregular whitish areas and erythematous regions to plaques, ulcerations, and crusts in more advanced cases. In addition, blurring of the vermilion border and changes in tissue elasticity are common signs. The disease affects fair‐skinned men, especially those with intense and prolonged exposure to the sun, such as outdoor workers. The lower lip is the most affected area due to its greater direct exposure to sunlight. However, the chronic and asymptomatic nature of AC often leads to its confusion with normal signs of aging, delaying diagnosis and favoring progression to LSCC. The rate of malignant transformation varies from 10% to 30%, and there are no clearly defined clinical or histopathological criteria for predicting individual risk [1].

Given this scenario, innovative strategies are needed to improve the early diagnosis of LSCC and the monitoring of AC. Artificial intelligence (AI) models have shown promise in the analysis of oral lesions, helping to predict and stratify the risk of malignancy. However, while machine learning (ML) techniques have advanced considerably for classifying lip lesions, there remains a notable gap in the literature regarding studies specifically focused on image segmentation. This task enables the precise delineation of affected areas, supporting both quantitative and qualitative lesion analysis. The scarcity of research addressing this step limits the development of automated tools that could offer noninvasive, objective, and low‐cost methods for a more detailed assessment of morphological patterns, while also aiding clinicians in large‐scale and diverse clinical settings [2, 3, 4].

This work is based on the Checklist for AI in Medical Imaging (CLAIM) [5], and the primary objective of this study was to develop and externally validate a deep learning (DL)–based segmentation model for the automatic delineation of AC and LSCC in clinical photographs. By providing accurate and robust lesion segmentation, the proposed model aims to support the development of AI‐assisted tools for lesion assessment, disease monitoring, and clinical decision‐making in oral medicine.

## 2 Methods

### 2.1 Ethical and Regulatory Compliance

All procedures were carried out in accordance with the Declaration of Helsinki. Ethical approval was obtained from the Piracicaba Dental School Ethics Committee on December 21, 2023 (Registration Number: 71009323.9.3004.5505), which also included the Federal University of Minas Gerais as a co‐participant, and separately from the Ethics Committee of the Federal University of Paraíba (Registration No. 72314323.0.0000.5188). Material Transfer Agreements were signed between the co‐participating institutions to permit sharing of clinical photographs and digital slides. No written consent has been obtained from the patients, as there is no patient‐identifiable data included.

### 2.2 Datasets

This retrospective cross‐sectional study developed a segmentation model using a dataset of 489 clinical photographs from 475 patients who attended the Oral Diagnostic Service (OROCENTRO) of the Piracicaba Dental School, State University of Campinas (FOP‐UNICAMP), Piracicaba, São Paulo, Brazil. An independent dataset for external validation included 42 images from patients at the Federal University of Paraíba (UFPB), João Pessoa, Paraíba, and 40 images from patients at the Federal University of Minas Gerais (UFMG), Belo Horizonte, Minas Gerais.

Inclusion criteria comprised individuals aged 18 years or older with clinical lesions on the lip vermilion border diagnosed as AC or LSCC, regardless of gender or skin color, suggesting a relative demographic heterogeneity in the sample. Unlike LSCC cases (all biopsy‐confirmed), most AC patients did not undergo an initial biopsy, as the clinical protocol mandates a follow‐up period before biopsy, which is only recommended when chronicity or concerning features are present. Therefore, absence of an initial biopsy was not an exclusion criterion for AC. Histopathological slides were reviewed by I.J.C.N., C.S.S., and A.L.D.A. and subsequently reassessed by P.A.V.; the external dataset was reviewed by P.R.F.B., F.P.F., and N.R.G., following World Health Organization diagnostic guidelines. To increase data variability, high‐quality photographs captured with either mobile phones or professional cameras were eligible.

Exclusion criteria included poor‐quality clinical photographs (e.g., inadequate lighting, blur, incomplete lesion visualization), lack of clinicopathological correlation (e.g., clinical suspicion of LSCC but whole‐slide images showing only oral epithelial dysplasia), LSCC cases lacking a biopsy for any reason (such as patient dropout), and photographs of other lip lesions unrelated to AC or LSCC.

In the FOP‐UNICAMP dataset, 14 patients had more than one photograph taken at different time points, with a minimum interval of 6 months; some of these images belonged to the same diagnostic class, while others represented different classes. Patients were split into training (80%), validation (10%; hyperparameter tuning), and testing (10%; model generalization) subsets. Individuals with multiple photographs were allocated exclusively to the training subset to minimize data leakage and prevent the convolutional neural network (CNN) from memorizing patient‐specific features. The split was performed nonrandomly, preserving the original class proportions, which were naturally imbalanced. In the external validation dataset, each patient contributed a single image (Table 1).

**TABLE 1:** Datasets.

Classes	Model development (FOP‐UNICAMP)					External validation	
						UFPB	UFMG
	Patients (*n*)	Images (*n*)	Training (80%)	Validation (10%)	Test (10%)	Images (*n*)	Images (*n*)
AC	390^a^	397	319	39	39	33	31
LSCC	80^a^	92	74	9	9	9	9
Total	475	489	393	48	48	42	40

Abbreviations: AC, actinic cheilitis; LSCC, lip squamous cell carcinoma.

^a^ Fourteen patients had more than one photo, sometimes with both photos belonging to the same class (AC = four patients with two photos, one patient with three photos; LSCC = two patients with two photos), and sometimes with photos belonging to distinct classes (seven patients with two photos). The external datasets comprised a single photograph per patient.

### 2.3 Ground Truth Masks (Manual Segmentation)

Clinical photographs were manually segmented by two clinicians (I.J.C.N. and H.A.R.) using the Aperio ImageScope visualization software (Leica Biosystems, Wetzlar, Germany) and the Huion Inspiroy H1060P graphics tablet. The segmentation target comprised the entire clinically affected lip region. For AC, which typically manifests as diffuse vermilion alterations, the entire area exhibiting visible clinical changes was included in the ground truth mask. For LSCC, the ground truth mask encompassed both the gross tumor boundaries and any adjacent diffuse alterations (e.g., concomitant AC). This annotation strategy ensured that the model learned to segment the entire diseased lip region rather than disease‐specific visual patterns. To reduce interobserver variability, a third clinician (A.L.D.A.) independently reviewed all ground truth masks, and any discrepancies were resolved by consensus among the three clinicians. Following this quality control step, each image was associated with an XML file containing the coordinates of the segmentation mask, which were subsequently used for model training and evaluation.

### 2.4 Architecture

The segmentation architecture used in this study, the EffiSegNet‐B0 model [6], was implemented using transfer learning. Inspired by Half‐U‐Net architectures [4], EffiSegNet‐B0 uses a pretrained EfficientNet B0 network with ImageNet weights [7] as an encoder. From this base, the feature maps of all the stages are summed and passed through a pair of ghost modules [8], which generate activation maps for composing the output in a computationally efficient way.

### 2.5 Preprocessing

The dimensions of the photographs ranged from 390 × 250 pixels to 6000 × 4000 pixels. All input images were resized to a square format, with the target size obtained directly from the EfficientNet API using a function that returns the model's expected input dimensions. Resizing employed Lanczos interpolation [9]. Pixel values were normalized using the ImageNet dataset's mean and standard deviation [7]. This transformation causes no visible change to the images, only applying standard micro‐adjustments for DL, as most architectures are pretrained on ImageNet. Due to the specific nature of the images, where transformations such as vertical flips or changes in scale could compromise the anatomical relationship of the elements, no data augmentation techniques were applied.

### 2.6 Training

To guarantee the model's reproducibility and comparability, we kept the parameters originally defined by Vezakis et al. [6]. The model was initialized with pretrained weights from the ImageNet dataset [7] and refined over 100 training epochs, with a batch size of 8. The loss function used was DiceCELoss from the *MONAI 1.4.0* library [10], and the optimizer used was Adam with decoupled weight decay regularization [11]. The learning rate varied from 10^−4^ to 10^−5^ during training, using the cosine annealing technique. The model's raw output was processed using the sigmoid activation function, producing a binary activation mask. The training was conducted using the PyTorch 2.5.1 framework [12] with acceleration on an NVIDIA GeForce GTX 1060 6GB GPU.

### 2.7 Evaluation

Once the model was trained, the test set was evaluated using the fully trained model. *PyTorch Lightning* was used as the framework for evaluation. Each predicted mask was compared with its respective ground truth mask, and the following metrics were calculated on a per‐image basis: accuracy, sensitivity (recall), precision, intersection over union (IoU), and Dice similarity coefficient (DSC) [13]. Dice and IoU were calculated for the positive class (lesion regions). For each metric, the mean, standard deviations, and 95% confidence intervals were computed using the Student's *t*‐distribution over the set of image‐wise metric values.

DSC and IoU were considered the primary metrics for evaluating lesion segmentation performance, while overall accuracy was interpreted with caution, given that pixel‐wise medical image segmentation tasks typically contain a large proportion of background pixels. Unlike accuracy, which can be artificially inflated by correctly classified background areas (especially when the lesion occupies only a small region), both DSC and IoU directly quantify the spatial overlap between the predicted mask and the ground truth mask. DSC is particularly well‐suited for imbalanced segmentation tasks as it balances sensitivity to false positives and false negatives, whereas IoU provides a stricter, complementary measure of overlap. Together, they offer a robust and established framework for clinically meaningful evaluation.

In addition, visual results were generated by comparing the input image, the ground truth mask, the raw model output, and the final predicted mask. To improve visual interpretability of the segmentation outputs, post‐processing operations were applied exclusively to the masks presented in qualitative result figures. The post‐processing pipeline consisted of bilinear upsampling, Gaussian filtering, and morphological smoothing with elliptical kernels. All quantitative analyses were performed using the original binary predictions generated by the network. The methodological workflow is shown in Figure 1.

**FIGURE 1:**
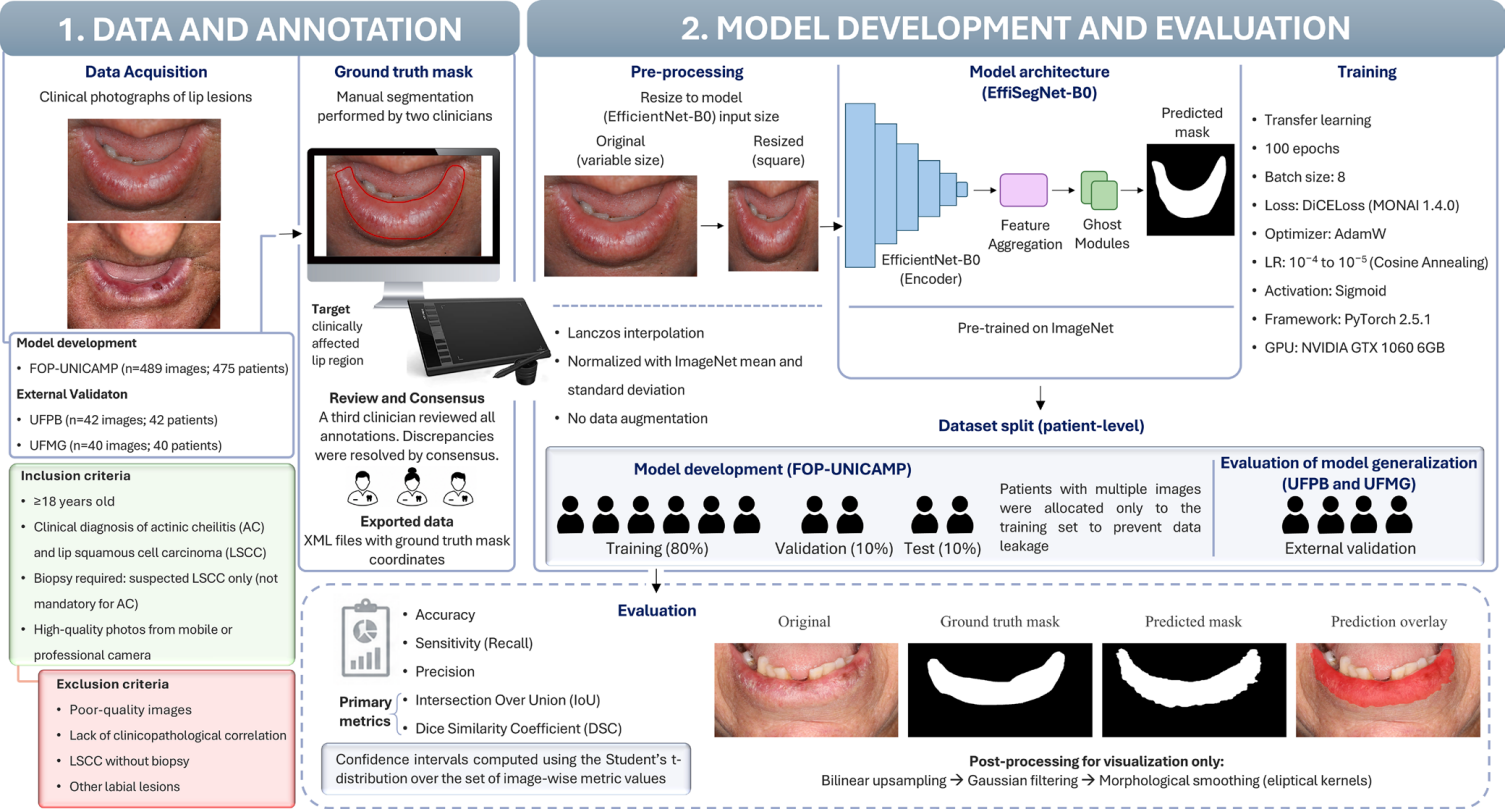
Methodological workflow. Schematic representation of the proposed study design and segmentation procedure.

## 3 Results

Table 2 presents the segmentation performance of the proposed model, which maintained a high segmentation accuracy above 95% on the internal test with no decline observed on the external validation datasets, providing evidence of good generalizability. Recall values improved from 0.89 on the internal test to 0.93 on the external validation, while precision declined from 0.89 to 0.83. The observed pattern (higher recall but lower precision on external validation) collectively underscores a trade‐off between sensitivity and precision, where the model becomes more inclusive (fewer false negatives) at the cost of more false positives. This suggests that while the model generalizes well in terms of detecting true positives, it may be slightly oversensitive to structures or noise in the external datasets.

**TABLE 2:** Comparison of the segmentation results of the EffiSegNet‐B0 model in internal test and external validation cohorts and Vezakis's work.

Metrics	Internal test dataset derived from FOP‐UNICAMP	External validation dataset (UFPB and UFMG)	KVasir‐SEG dataset [6]
Accuracy	0.956 ± 0.026 [0.949–0.964]	0.955 ± 0.023 [0.950–0.960]	—
Sensitivity	0.894 ± 0.076 [0.872–0.916]	0.934 ± 0.049 [0.923–0.945]	0.9368
Precision	0.891 ± 0.089 [0.865–0.916]	0.833 ± 0.105 [0.810–0.856]	0.9475
IoU	0.802 ± 0.088 [0.777–0.828]	0.782 ± 0.087 [0.763–0.801]	0.8794
Dice	0.888 ± 0.057 [0.871–0.904]	0.875 ± 0.060 [0.862–0.888]	0.9304

*Note:* Values are reported as mean ± standard deviation, with 95% confidence intervals shown in brackets [lower, upper].

Abbreviation: IoU, intersection over union.

The Dice coefficient of 0.87 on the external validation represents excellent overlap between the predicted segmentation and the ground truth, meaning the model makes few errors in delineating the structures of interest. The slight IoU drop from 0.80 to 0.78 is marginal (only 2 percentage points), which implies that the reduction in overlap accuracy is minimal and likely due to natural variations between datasets (e.g., differences in image acquisition, minor anatomical variations, or noise). In practice, these results indicate that the model remains reliable and clinically useful even when applied to new, external data.

When comparing the metrics obtained in our experiments with those reported by Vezakis et al. [6] on the Kvasir‐SEG dataset, our model showed a reduction in the Dice coefficient of approximately 6% (from 0.9304 to a mean of 0.875) and a reduction in IoU of about 11% (from 0.8794 to 0.782). However, this is not a direct comparison, as the two datasets represent distinct segmentation tasks: Kvasir‐SEG focuses on gastrointestinal polyps, whereas our dataset comprises lip lesions, which often present with more diffuse boundaries and greater morphological variability. Considering these differences, the observed performance gap is relatively modest.

Furthermore, the strong performance of the proposed study can be visually confirmed by the prediction masks generated from external validation (Figure 2). The model remains robust across different lesion categories, showing no decline in segmentation performance despite drastic visual variations, such as ulceration, crusting, or keratinization. This robustness suggests that the model is forced to focus on learning the relevant anatomical region rather than disease‐specific textures. Consequently, the model consistently extracts a complete region of interest, regardless of the lesion phenotype.

**FIGURE 2:**
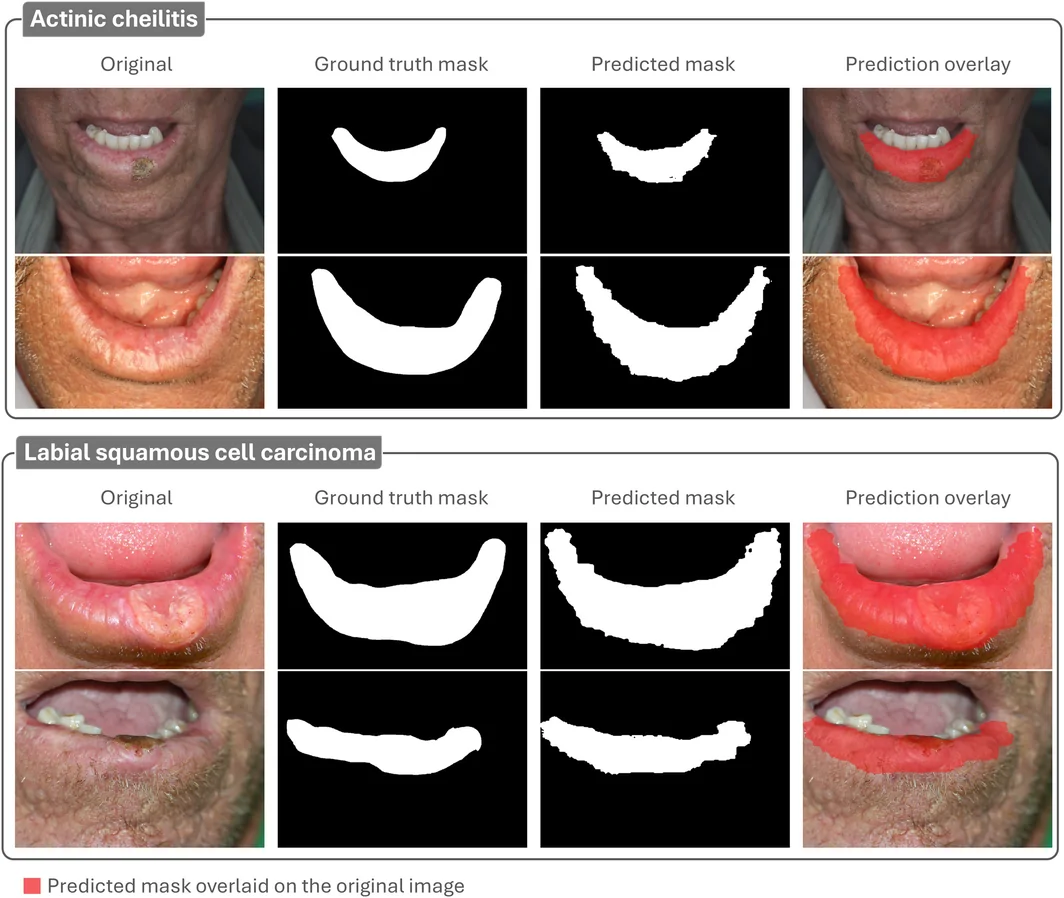
Representative qualitative segmentation results for AC and LSCC. Examples of clinical photographs used for model evaluation are shown alongside the corresponding ground truth masks, predicted segmentation masks, and prediction overlays on the original images.

## 4 Discussion

To date, no study has focused on the automatic segmentation of lip lesions. While classification models can distinguish between differential diagnoses, they do not provide spatial information about lesion boundaries or extent. Segmentation offers a finer‐grained output, which is essential for monitoring disease progression, planning biopsies, or guiding surgical margins. Therefore, the present study proposes a DL‐based segmentation model specifically designed for lip lesions, using a custom‐trained EffiSegNet‐B0 architecture. The model was developed and validated on a large dataset of clinical photographs from multiple institutions, with ground truth masks covering the entire diseased lip territory. To the best of our knowledge, this is the first study to address automatic segmentation of lip lesions, filling a critical gap between computer‐aided diagnosis and clinically actionable spatial delineation.

To date, there have only been three previous studies focusing on lip lesion classification using approaches based on AI for diagnostic support. In a preliminary study, Paulino et al. [2] investigated the use of a hybrid classifier, combining SVM and KNN, to diagnose lip lesions in thermal images. The research developed a method for classifying patient images into three groups: healthy, squamous cell carcinoma, and AC. The KNN reached 100% accuracy, sensitivity, and precision for differentiating AC and LSCC, demonstrating the model's potential efficiency as a tool to aid diagnosis, despite the limited sample size. Spyridonos et al. [3] developed a probabilistic model to diagnose AC using digital photographs. The methodology involved extracting morphological descriptors from the vermilion border of the lip and quantifying retraction and irregularity through fractal features and Fourier descriptors. A relevance vector machine (RVM) algorithm was used to build a classification model that achieved high sensitivity (94.6%) and specificity (96%). The study demonstrates the feasibility of an objective, noninvasive, and low‐cost method for monitoring AC in diverse clinical settings, with implications for large‐scale epidemiological and interventional studies. More recently, another study by Liu and Bagi proposed a custom‐designed 19‐layer CNN for the automated diagnosis of oral cancer using clinical images of the lips and tongue [14]. The proposed CNN achieved an accuracy of 99.54%, sensitivity of 95.73%, specificity of 96.21%, precision of 96.34%, and *F*_1_ score of 96.03%, outperforming transfer learning benchmarks such as SqueezeNet, AlexNet, Inception, VGG19, and ResNet50 under identical experimental conditions. The study demonstrated that a lightweight, custom architecture can yield high diagnostic performance, supporting its potential for real‐world clinical application.

Automatic segmentation of medical images has been widely explored in recent years because it can support image analysis workflows and contribute to the development of computer‐assisted diagnostic tools. Although robust architectures such as U‐Net are widely adopted, their high computational cost restricts their use in scenarios with technological limitations. Lightweight architectures, such as EffiSegNet, present a promising alternative by balancing efficiency and competitive segmentation performance. However, to date, its application has been limited to the Kvasir‐SEG dataset for gastrointestinal tasks [6], leaving uncertainties regarding its generalization to other contexts, such as the segmentation of lip lesions. Therefore, the application of an EffiSegNet model, quite recent in the area of medical image segmentation, to a new dataset brings interesting contributions such as (i) the external validation of the model, expanding evidence on its versatility and generalization capacity in different clinical contexts, and (ii) the comparative analysis, allowing us to identify differences in performance between datasets and their distinctive characteristics.

Unlike the original study with intestinal polyps [6], this work opted not to use augmentation transformations due to the specific nature of the images used, since the structures of interest can be in quite different portions of the images in terms of position and rotation. For lip lesions, mostly found on the lower lip, transformations such as the vertical flip and changes in scale and elasticity can compromise the anatomical relationship within the images. This methodological choice highlights that each DL approach should be adjusted according to the data and targeted for the intended application.

Our approach yielded strong performance results, providing evidence that the model is robust, generalizes well, and can be safely deployed in new scenarios without significant loss of quality. However, two aspects regarding the metrics caught our attention: the precision drop on external validation and the trade‐off between recall and precision. The precision drop on external validation likely reflects differences in image acquisition and annotation between institutions. The external ground truth was established by a different reviewer team, and the lack of data augmentation may limit the model's ability to generalize to unseen visual variations. Although precision decreased, the high recall suggests the model remains clinically safe by rarely missing true lesions. From a clinical perspective, the high recall (0.93) ensures that few lesions are missed, which is desirable for screening and margin delineation. The moderate precision (0.83) may lead to false positives, potentially resulting in unnecessary biopsies, especially for AC. However, this trade‐off is acceptable for LSCC, where missing a lesion carries higher risk. Threshold adjustment can tailor the model's behavior to specific clinical scenarios.

This study has inherent limitations due to its retrospective and cross‐sectional design, which restricts causal inferences and may introduce selection bias. The data split was performed nonrandomly, preserving the natural class imbalance, which may affect performance for the less represented class. No data augmentation techniques were applied because geometric transformations could compromise anatomical relationships, but this also reduces training variability. Although allocating multiple images from the same patient exclusively to the training set prevents data leakage, the test set and external validation do not capture intra‐patient variability. Manual annotation of the regions of interest, although reviewed by consensus, is inherently subjective, especially for diffuse lesions such as AC. Moreover, most AC cases did not have histopathological confirmation by biopsy, relying solely on clinical criteria. External validation was restricted to two Brazilian institutions, limiting generalization to other populations and image acquisition conditions. The wide variation in photograph resolution (390 × 250 to 6000 × 4000 pixels) and resizing to a square format may impact performance on low‐resolution images. Finally, post‐processing was applied exclusively to the masks presented in qualitative figures, which may make the visual examples appear smoother than the raw quantitative results, although numerical analyses used the original binary predictions.

## 5 Conclusion

This is the first study to automate lip lesion segmentation, offering a foundation for clinically actionable AI tools in oral medicine. The EffiSegNet‐B0 model demonstrated robust and generalizable segmentation of lip lesions (AC and LSCC) on internal and external datasets. A trade‐off between high recall and moderate precision on external validation suggests the model is sensitive but prone to false positives, likely due to cross‐institutional differences in image acquisition and annotation. Clinically, the high recall supports screening and margin delineation, especially for LSCC, while moderate precision may lead to unnecessary biopsies for diffuse AC. Future work should address this trade‐off through threshold adjustment or institution‐specific fine‐tuning. Furthermore, a comparative analysis between the performance of the proposed model and a classic U‐Net or other models in the EffiSegNet family in the same context could provide interesting insights into the cost that EffiSegNet's efficiency brings in terms of segmentation quality.

## Author Contributions

**Vitor Simbalista Teixeira Soares:** conceptualization, writing – original draft. **Anna Luíza Damaceno Araújo:** conceptualization, supervision, project administration, data curation, writing – original draft. **Ivan José Correia‐Neto:** conceptualization, data curation, writing – original draft. **Matheus Cardoso Moraes:** supervision, project administration, methodology (model development), software, formal analysis, validation, writing – review and editing. **Marcio Ajudarte Lopes:** supervision, project administration, writing – review and editing. **Cristina Saldivia‐Siracusa:** data curation. **Raísa Sales de Sá:** data curation. **Hugo Abritta Reis:** data curation. **Paulo Rogério Ferreti Bonan:** data curation. **Nathalia Rodrigues Gomes:** data curation. **Felipe Paiva Fonseca:** data curation. **Ayrton da Costa Ganem Filho:** writing – review and editing. **André Carlos Ponce de Leon Ferreira de Carvalho:** writing – review and editing. **Thierry Urruty:** writing – review and editing. **Pablo Agustin Vargas:** writing – review and editing. **Alan Roger Santos‐Silva:** writing – review and editing. **Luiz Paulo Kowalski:** writing – review and editing. All authors have read and agreed to the published version of the manuscript.

## Funding

This study was financed, in part, by the São Paulo Research Foundation (FAPESP), Brazil. Process Numbers #2021/14585‐7 and #2022/13069‐8. Matheus Cardoso Moraes is a postdoctoral researcher who holds two incentives funded by the National Council for Scientific and Technological Development (CNPq, Brazil), with process numbers 443992/2023‐1 and 406947/2023‐6, dedicated to the renovation of computers in the Biomedical Image Processing Laboratory. Felipe Paiva Fonseca holds incentives funded by The Minas Gerais Research Foundation (FAPEMIG).

## Ethics Statement

This study was performed in accordance with the Declaration of Helsinki and was approved by the Piracicaba Dental School Ethical Committee on December 21, 2023 (Registration number: 71009323.9.3004.5505) and by the Federal University of Paraíba Ethical Committee (Registration number: 72314323.0.0000.5188), which also included Material Transfer Agreements between the co‐participant institutions for image sharing.

## Conflicts of Interest

The authors declare no conflicts of interest.

## Data Availability

The datasets used and/or analyzed in this study are available from the corresponding author upon reasonable request. All authors agree to be accountable for all aspects of the work and will ensure that any questions regarding the accuracy or integrity of any part of the work are appropriately investigated and resolved.
